# Sun Stroke

**Published:** 1869-02-15

**Authors:** M. Waterhouse

**Affiliations:** Portage City, Wis.


### *Sun Stroke.*

BY M. WATERHOUSE, M.D., PORTAGE CITY, WIS.

EDITORS JOURNAL:

From reading various articles in THE JOURNAL of the 16th December, upon the treatment of sun stroke, I am induced to communicate some of my own experience in that disorder. The theory of sun stroke seems to be laid down somewhat vaguely, as the effect of extreme heat on the nervous system in persons fatigued and of previously enfeeblee constitution. How far out of the way this definition may be, or what the particular indications for treatment would follow it, if any, I do not stop to inquire, but the remedies that have been tried are numerous and unsatisfactory, and in all ages and modes of treatment the head has invariably received the largest share of attention, notwithstanding that post-mortem examinations seldom furnish any evidence that the brain has suffered material injury, on the contrary, the lungs are nearly always found in a state of congestion.

My belief is, that in by far the greater proportion of cases, the effect of the heat is local and confined to the cervical and dorsal regions of the spinal cord. During the heated period of each summer, for a number of years past, I have seen a greater or less number of cases of sun stroke and noted particularly the mode of attack, and especially the position of the individual at the time, and so far as I can recollect, every person was so situated as to receive the rays of the sun more or less directly on the back, as for instance:

J. O., of Portage City, a painter by trade, aged about forty-five, of previously good health and strong constitution, was painting letters on the side of a railroad car with his back exposed to the direct rays of the sun, at about noon, suddenly felt his eyesight begin to fail, and in a few minutes fell down comatose, with slow, feeble pulse. Stertorous breathing and inability to swallow, and had a number of violent convulsions.

H. A., a carpenter, aged about thirty, a strong, healthy, vigorous man, was nailing siding on a house, with his back to the sun, at about two o'clock, P. M., suddenly fell down comatose, but no convulsions.

J. M., a farmer, aged thirty-two, of previously good health and constitution, was binding wheat in the harvest field in a stooping position, so that which ever way he was facing he received the rays of the sun on his back, and was attacked with coma and fell down at 3 o'clock, P. M., had no convulsions nor stertorous breathing.

Another man, whose previous health and habits I did not learn, was journeying through the country in a wagon, and sitting with his back to the sun, and could not have been very greatly fatigued, was seized with sun stroke and fell over in his wagon with coma, and more or less convulsions.

These cases, and about a dozen others that have fallen under my observation, all being attacked in similar positions and circumstances, were all treated alike, viz; by cups applied to the cervical and dorsal sections of the spine, followed with ice-bags to the same regions, and in every case with relief more or less promptly, and final recovery with *very little* other treatment. I have also noticed in examinations of invalid pensioners, whose disability originate in sun stroke, that besides being generally debilitated they have all more or less spinal tenderness, and some of them have habitual convulsions.

In regard to the lesions found in the lungs after death from sun stroke is it not probable that the cause is to be found in the effects of extreme heat upon those nervous centres that are most directly concerned in the function of respiration.

Now all this may nothing new to many of your readers, nor do I suppose that there is sufficient in the limited number of cases that I have seen to establish any rule that will apply to all cases, yet there seemed enough in them to impress me strongly with the idea of addressing my efforts to the spine, and to warrant me in giving you such thoughts as have occured to me on the subject.

